# Radiofrequency enhances drug release from responsive nanoflowers for hepatocellular carcinoma therapy

**DOI:** 10.3762/bjnano.15.49

**Published:** 2024-05-22

**Authors:** Yanyan Wen, Ningning Song, Yueyou Peng, Weiwei Wu, Qixiong Lin, Minjie Cui, Rongrong Li, Qiufeng Yu, Sixue Wu, Yongkang Liang, Wei Tian, Yanfeng Meng

**Affiliations:** 1 School of Public Health, Shanxi Medical University, Taiyuan, Shanxi 030001, China; 2 College of Medical Imaging, Shanxi Medical University, Taiyuan, Shanxi, 030001, China; 3 Department of Radiology, Taiyuan Central Hospital of Shanxi Medical University, Taiyuan, Shanxi 030009, China; 4 The Ninth Clinical Medical School of Shanxi Medical University, Taiyuan, Shanxi 030009, China; 5 Department of Medical Imaging, Changzhi Medical College, Changzhi, Shanxi, 046000, China; 6 Academy of Medical Sciences, Shanxi Medical University, Taiyuan, Shanxi 030001, China; 7 Department of General Surgery, Shanxi Cardiovascular Hospital, Taiyuan, Shanxi 030024, China

**Keywords:** curcumin, hepatocellular carcinoma, magnetic resonance imaging (MRI), radiofrequency (RF) hyperthermia, responsive nanoflower

## Abstract

Hepatocellular carcinoma (HCC) is the sixth most common malignant tumor and the third leading cause of cancer death worldwide. Most patients are diagnosed at an advanced stage, and systemic chemotherapy is the preferred treatment modality for advanced HCC. Curcumin (CUR) is a polyphenolic antineoplastic drug with low toxicity obtained from plants. However, its low bioavailability and poor solubility limit its functionality. In this study, radiofrequency- (RF) enhanced responsive nanoflowers (NFs), containing superparamagnetic ferric oxide nanoclusters (Fe_3_O_4_ NCs), – CUR layer, – and MnO_2_ (CUR-Fe@MnO_2_ NFs), were verified to have a thermal therapeutic effect. Transmission electron microscopy was used to characterize the CUR-Fe@MnO_2_ NFs, which appeared flower-like with a size of 96.27 nm. The in vitro experimental data showed that RF enhanced the degradation of CUR-Fe@MnO_2_ NFs to release Mn^2+^ and CUR. The cytotoxicity test results indicated that after RF heating, the CUR-Fe@MnO_2_ NFs significantly suppressed HCC cell proliferation. Moreover, CUR-Fe@MnO_2_ NFs were effective *T*_1_/*T*_2_ contrast agents for molecular magnetic resonance imaging due to the release of Mn^2+^ and Fe_3_O_4_ NCs.

## Introduction

Hepatocellular carcinoma (HCC) is the sixth most common malignant tumor and the third leading cause of cancer death worldwide. Furthermore, the incidence of HCC has been increasing [1]. Despite advancements in early diagnosis, a significant portion of HCC patients are still diagnosed at an advanced stage [2–3]. Atezolizumab combined with bevacizumab is the primary recommended systematic treatment for advanced HCC according to mainstream guidelines [4–5]. Even if these drugs exhibit strong targeting and notable therapeutic effects, their efficacy is limited by their immunogenicity, and their application is limited by high costs [6]. In addition to the aforementioned treatments, systemic chemotherapy drugs including sorafenib and lenvatinib have been shown to be effective at improving overall survival. Nonetheless, patients often discontinue these treatments due to significant side effects such as hypertension, proteinuria, and skin toxicity [7–8]. Hence, there is a pressing need to develop new therapeutic modalities that offer substantial efficacy while minimizing side effects.

Extensive efforts have been dedicated to drug development and delivery technologies in pursuit of enhanced therapeutic effects and reduced side effects [9]. Among these, curcumin (CUR), a natural plant-derived polyphenolic drug, has garnered considerable attention due to its potential in treating HCC [10–13]. Curcumin can promote HCC cell apoptosis by activating p38, a cancer suppressor gene [14]. Curcumin can also curtail HCC angiogenesis by decreasing the expression of vascular endothelial growth factors (VEGFs) [15]. Furthermore, CUR has the potential to inhibit HCC by reducing the number of myeloid-derived suppressor cells (MDSCs) and interfering with angiogenesis by downregulating the expression of VEGFs and the endothelial cell adhesion molecule CD31 [16]. However, disadvantages of CUR include its poor stability, rapid metabolism, and low solubility, which limits its application [17–20].

To address the aforementioned challenges, intelligent delivery systems have been developed based on the abnormal physiological signals in the tumor microenvironment (TME), such as a low pH, high glutathione (GSH) levels, hypoxia, and the expression of specific enzymes [21]. Such intelligent nanoparticles (NPs) have successfully improved the solubility and distribution of CUR through the enhanced permeability and retention (EPR) effect, thereby extending the drug circulation time and improving its accumulation and effective release within tumors [9,22–23]. The newly developed class of nanoparticles with a structure similar to that of plant flowers is called nanoflowers (NFs). The special structure of nanoflowers improves the stability and efficiency of the surface reaction [24]. Furthermore, prior research has verified that radiofrequency (RF) hyperthermia can significantly improve the sensitivity of cancer cells to chemotherapy at approximately 42 °C [25–27]. Radiofrequency-induced hyperthermia has been confirmed to augment the permeability of the plasma membrane, facilitating the entry of drugs into tumor cells to kill them [28–29].

In this study, we present the synthesis of an intelligent TME-responsive nanomaterial, superparamagnetic ferric oxide nanoclusters (Fe_3_O_4_ NCs), – CUR layer, – and MnO_2_ (CUR-Fe@MnO_2_ NFs). These NFs carry CUR and Fe_3_O_4_ NCs, achieve sustained and concurrent drug release, and can be used for molecular magnetic resonance imaging (MRI). Moreover, we explored the ability of the NFs to release drugs and evaluated their cytotoxic effects when combined with RF hyperthermia. Using these CUR-Fe@MnO_2_ NFs combined with RF, hyperthermia is a candidate method for the targeted treatment of HCC via combined chemotherapy/hyperthermia.

## Results and Discussion

### Characterization

**Figure 1:**
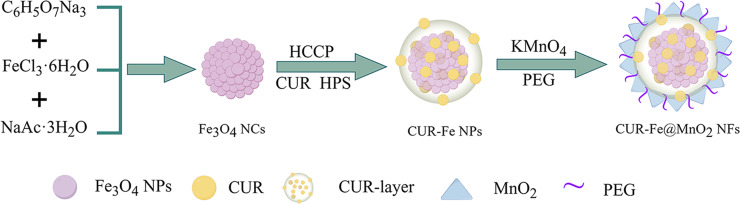
Synthesis of the Fe_3_O_4_ NCs, CUR-Fe NPs, and CUR-Fe@MnO_2_ NFs. Figure 1 was drawn using Figdraw (https://www.figdraw.com), export ID AOPIS34314. The materials contained in the image are copyrighted by Home for Researchers. This content is not subject to CC BY 4.0.

**Figure 2:**
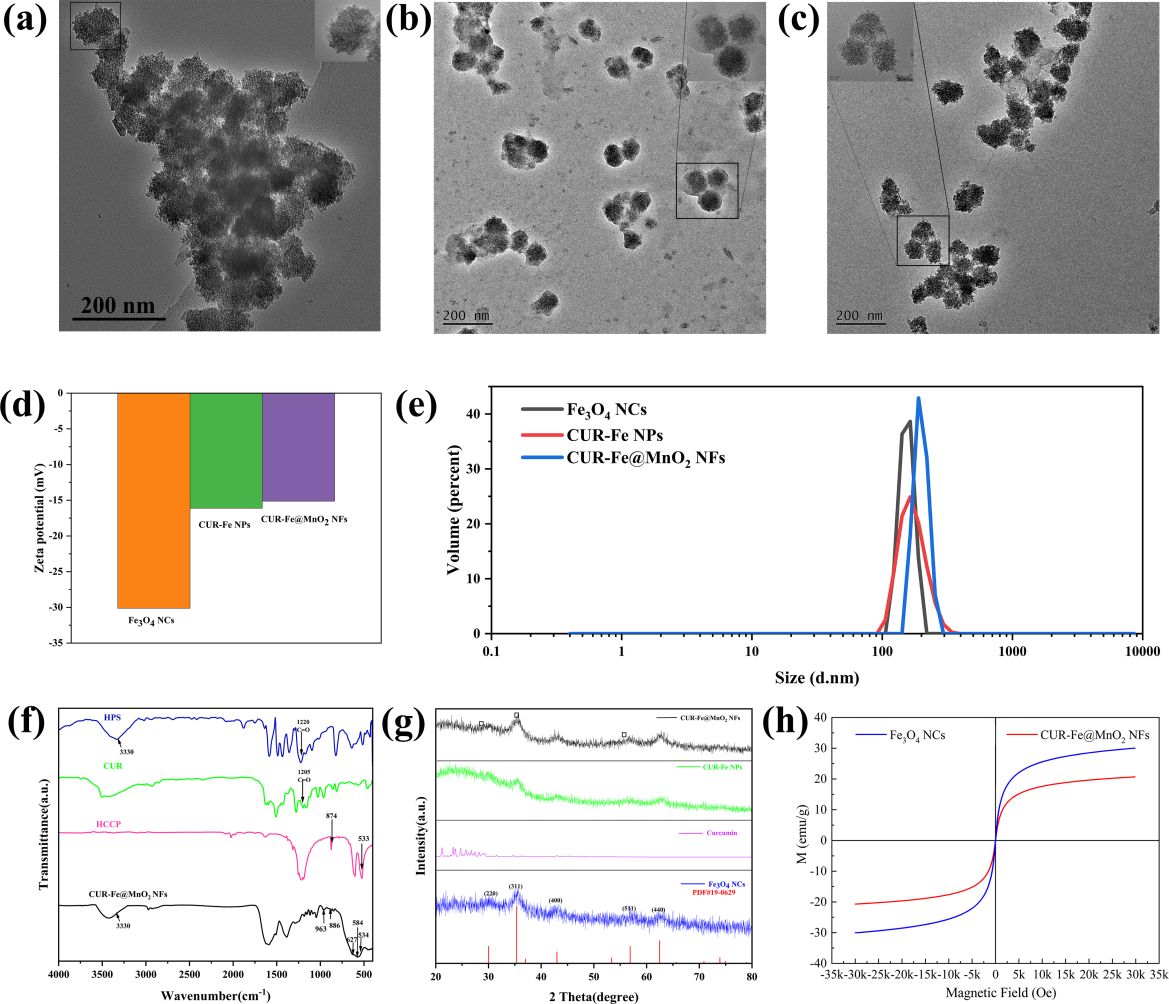
Characterization of the nanomaterials. a–c) TEM images of the Fe_3_O_4_ NCs, CUR-Fe NPs, and CUR-Fe@MnO_2_ NFs (scale bar: 200 nm). d,e) Zeta potentials and hydrodynamic diameters of the Fe_3_O_4_ NCs, CUR-Fe NPs, and CUR-Fe@MnO_2_ NFs (the polydispersity index values were 0.184, 0.260, 0.269, respectively). f) FTIR spectra of HCCP, CUR, HPS, and the CUR-Fe@MnO_2_ NFs. g) XRD patterns of the Fe_3_O_4_ NCs, curcumin, CUR-Fe NPs, and CUR-Fe@MnO_2_ NFs. h) Hysteresis loop of the Fe_3_O_4_ NCs and CUR-Fe@MnO_2_ NFs, indicating that they were superparamagnetic.

The Fe_3_O_4_ NCs were prepared by a microwave hydrothermal synthesis method and coassembled with a CUR layer on their surface. Then, CUR-Fe@MnO_2_ NFs were obtained by further modification with MnO_2_ and polyethylene glycol (PEG), which increased the stability and dispersion of the CUR-Fe@MnO_2_ NFs (Figure 1). MnO_2_ is difficult to degrade under physiological conditions in vivo. The presence of MnO_2_ protects the drug layer and reduces the loss of drugs to circulation. In tumors, MnO_2_ were degraded to produce Mn^2+^ and oxygen by response TME, exposing the drug layer for drug release and to exert antitumor effects. At the same time, Mn^2+^ can act as an MRI contrast agent. Oxygen can alleviate tumor hypoxia and regulate TME to improve antitumor efficiency. In addition, PEG-modified NFs may significantly enhance passive targeting and retention via the EPR effect, thus enhancing their efficacy in cancer treatment [30]. The Fe_3_O_4_ NCs, Fe_3_O_4_ NCs-CUR layer nanoparticles (CUR-Fe NPs), and CUR-Fe@MnO_2_ NFs were observed via transmission electron microscopy (TEM) (Figure 2a–c). The sizes of Fe_3_O_4_ NCs, CUR-Fe NPs, and CUR-Fe@MnO_2_ NFs were 50.72 ± 10.16 nm, 94.00 ± 12.21 nm, and 96.27 ± 19.14 nm, respectively. The Fe_3_O_4_ NCs surface coating can be seen in Figure 1b, indicating that CUR was successfully incorporated. CUR-Fe@MnO_2_ NFs appeared flower-like in the TEM images, indicating successful modification with MnO_2_. The zeta potentials of the Fe_3_O_4_ NCs, CUR-Fe NPs, and CUR-Fe@MnO_2_ NFs were −30.133 mV, −16.133 mV, and −15.133 mV, respectively (Figure 2d) and the hydrodynamic diameters were 156 nm, 177 nm, and 199 nm, respectively (Figure 2e). The average size obtained from TEM was different from the sizes found by dynamic light scattering (DLS). The reason is that the TEM image depicts the size of the sample in the dry state, while the DLS method depicts the size of the hydrated state [31]. The DLS depicted size is the closest to that in vivo.

The Fourier-transform infrared (FTIR) spectrum of CUR exhibited vibrational absorption peaks at 1207 cm^−1^ and 3330 cm^−1^ corresponding to C=O and –OH, respectively, in the phenolic hydroxy group (Figure 2f). Similarly, for bis(4-hydroxyphenyl) disulfide (HPS), the absorption peaks corresponding to the C=O and –OH groups of the phenolic hydroxy group were located at 1220 cm^−1^ and 3330 cm^−1^, respectively. Furthermore, the peaks at 533 cm^−1^ and 620 cm^−1^ corresponded to the P–Cl vibrations of the hexachlorocyclotriphosphazene (HCCP) molecule, while the peaks at 874 cm^−1^ and 1217.3 cm^−1^ resulted from the vibrations of P–N and P=N in HCCP. The FTIR spectrum of the CUR-Fe@MnO_2_ NFs revealed an absorption peak corresponding to the Fe–O bond in the Fe_3_O_4_ NCs at 584 cm^−1^. A new absorption peak at 963 cm^−1^ indicated the formation of P–O– CUR/HPS bonds, while the intensities of the peaks at 534 cm^−1^, 627 cm^−1^, and 874 cm^−1^ corresponded to P–Cl and P–N on HCCP, respectively. The strong absorption band at 3330 cm^−1^ in the NFs spectrum attributed to the O–H bond of CUR/HPS decreased, suggesting that the chlorine atom of P–Cl in HCCP was substituted by O–H. Additionally, other characteristic absorption peaks of CUR and HPS were retained. These findings confirmed the successful synthesis of CUR-Fe@MnO_2_ NFs.

According to the X-ray diffraction (XRD) pattern (Figure 2g), the 2θ diffraction peaks at 30.1° (220), 35.4° (311), 37.0° (222), 43.1° (400), 53.4° (422), 56.9° (511), and 62.7° (440) are consistent with the face-centered cubic structure of Fe_3_O_4_ (PDF#19-0629). The XRD pattern of CUR shows amorphous halos at about 20° to 30°. New broad amorphous halos of greater intensity appeared at approximately 20° to 30°, indicating the presence of a layer on CUR-Fe NPs and CUR-Fe@MnO_2_ NFs. Furthermore, the three new diffraction peaks at 28.7°, 37.34°, and 64.8° confirmed the presence of MnO_2_.

The magnetic properties of the NFs were verified by magnetic hysteresis loops. Figure 2h shows that Fe_3_O_4_ NCs and CUR-Fe@MnO_2_ NFs were superparamagnetic, and their magnetic saturation (Ms) reached 30.6 emu·g^−1^ and 20.7 emu·g^−1^, respectively. Compared with the Ms of Fe_3_O_4_ NCs, the Ms of CUR-Fe@MnO_2_ NFs significantly decreased, which indicated the successful introduction of the nonmagnetic CUR layer and MnO_2_.

### Relaxation rate measurements

**Figure 3:**
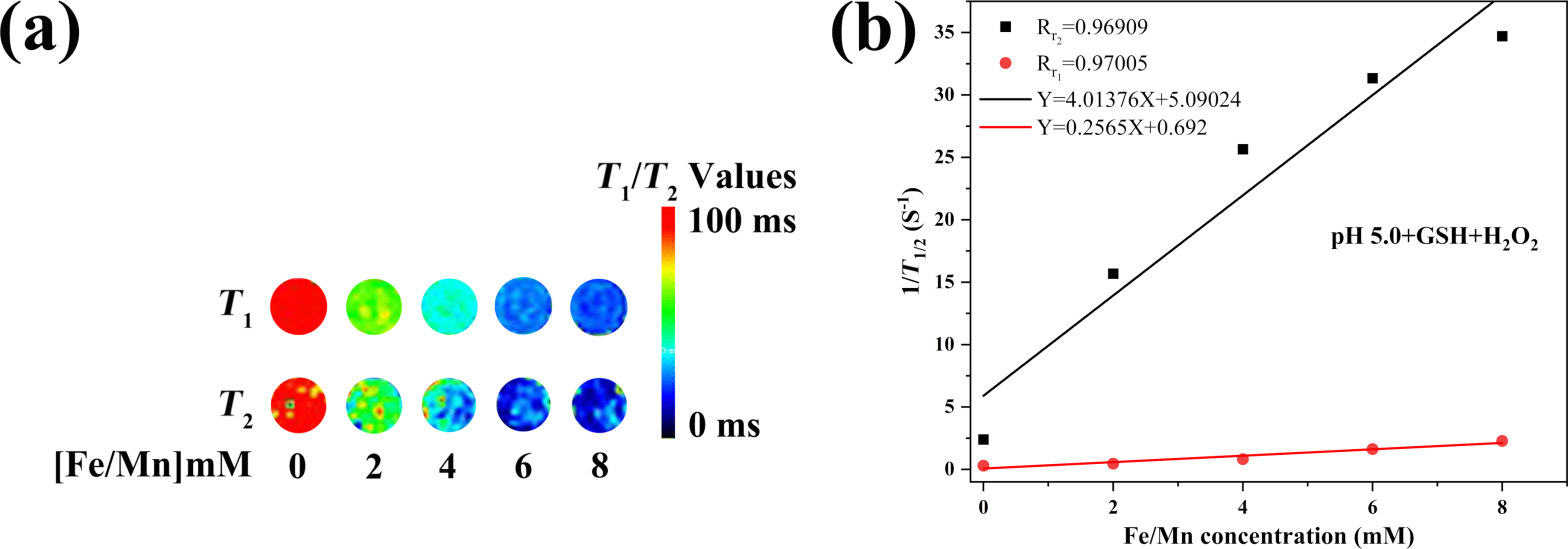
a) *T*_1_/*T*_2_-mapping MR imaging of CUR-Fe@MnO_2_ NFs at pH 5.0+GSH+H_2_O_2_. b) Longitudinal relaxation rate, *r*_1_ (red line), and transversal relaxation rate, *r*_2_ (black line) of CUR-Fe@MnO_2_.

CUR-Fe@MnO_2_ NFs can affect *T*_1_/*T*_2_ MRI contrast. After incubation with different concentrations of CUR-Fe@MnO_2_ NFs in a simulated TME for 24 h, the *T*_1_ value decreased in response to Mn^2+^ release, and the *T*_2_ value decreased in response to Fe_3_O_4_ (Figure 3a). The longitudinal and transverse relaxation rates of the NFs were *r*_1_ = 0.2565 mM^−1^·s^−1^ and *r*_2_ = 4.01376 mM^−1^·s^−1^, respectively (Figure 3b). Therefore, the CUR-Fe@MnO_2_ NFs showed marked sensitivity to the TME, suggesting that they are excellent dual-modal *T*_1_/*T*_2_ contrast agents.

### NFs degradation and drug release

**Figure 4:**
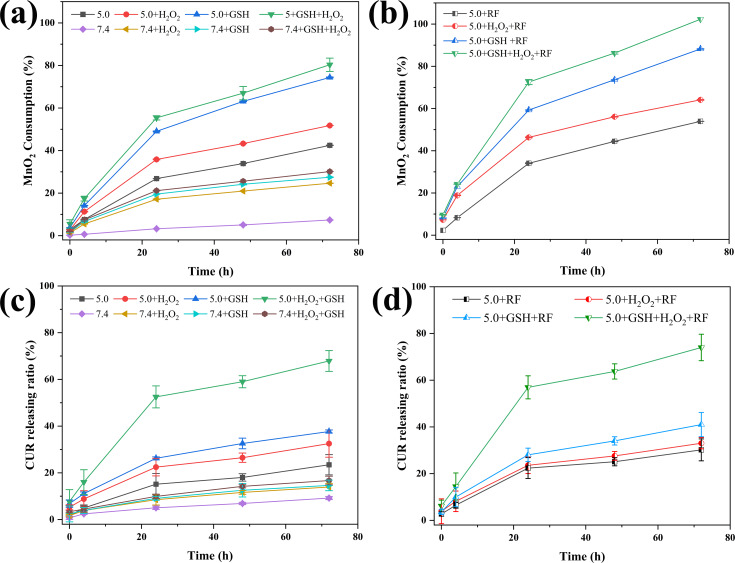
a) Curves depicting the degradation of MnO_2_ from CUR-Fe@MnO_2_ NFs in different environments. b) Curves depicting the degradation of MnO_2_ from the NFs after RF heating to 41 ± 1 °C for 20 min. c) Curves depicting the release of CUR from the NFs in different environments. d) Curves depicting the release of CUR from the NFs after RF heating to 41 ± 1 °C for 20 min.

CUR-Fe@MnO_2_ NFs can respond to a simulated TME by degrading MnO_2_ to release Mn^2+^ and lysing the CUR layer to release CUR. Mn^2+^ was completely released under the simulated TME condition by RF heating to 41 ± 1 °C for 20 min (Figure 4b). Up to 80% of the Mn^2+^ was released without RF heating. Additionally, only 7.3% of the Mn^2+^ was released at pH 7.4, but this percentage increased to 42% at pH 5.0 (Figure 4a). These results indicate that RF heating enhances degradation of NFs.

Due to their structure, CUR-Fe@MnO_2_ NFs efficiently increased the drug loading efficiency (DLE) of CUR and improved its stability. The drug encapsulation efficiency (DEE) and DLE of the CUR-Fe@MnO_2_ NFs were 57% and 70%, respectively. The drug release curves showed similar trends under different environmental conditions (Figure 4c,d). At pH 7.4, only 9% of CUR was released from the CUR-Fe@MnO_2_ NFs, indicating that CUR release is lower under physiological conditions. However, 68% of CUR was released in TME. The results showed that NFs is sensitive to TME and more CUR is released to exert an antitumor effect. Approximately 74% of CUR in the CUR-Fe@MnO_2_ NFs were released under simulated TME conditions by RF heating to 41 ± 1 °C for 20 min. Therefore, RF heating enhanced CUR release from CUR-Fe@MnO_2_ NFs in the simulated TME.

Drug release kinetics model fitting was also performed. The Higuchi, Ritger–Peppas, zero-order, and first-order methods were used to fit the experimental data, and *R*^2^ and release constants were calculated, as shown in Table 1. Among them, the Higuchi model has the highest degree of fitting with the release of NFs in different simulated environments within 72 h, which indicates conformity with the Fick diffusion mechanism. Meanwhile, we found that RF heating does not affect the release kinetics model. Ritger–Peppas is a semiempirical model, and n is an indicator of the drug release mechanism, where n ≤ 0.45 represents the Fick diffusion. The n values calculated in this study are all less than 0.45, indicating that the CUR release from NFs is in accordance with the Fick diffusion. Similarly, the Higuchi model has the highest degree of fitting with the release of CUR-Fe NPs and is in accordance with the Fick diffusion (Table 2).

**Table 1:** Comparison of CUR-Fe@MnO_2_ NFs release models in vitro.^a^

Groups	*R*^2^ value for release							

	Zero-order model	*K*_0_	First-order model	*k*_1_	Higuchi model	*k*_H_	Ritger–Peppas model	*k*

pH 7.4	0.96	0.11	0.94	0.03	0.99	0.97	0.98	1.09
pH 7.4 + H_2_O_2_	0.95	0.16	0.94	0.04	0.99	1.47	0.97	2.07
pH 7.4 + GSH	0.95	0.17	0.92	0.04	0.99	1.51	0.95	2.16
pH 7.4 + GSH + H_2_O_2_	0.97	0.19	0.89	0.04	0.98	1.67	0.92	2.33
pH 5.0	0.93	0.29	0.97	0.04	0.98	2.60	0.98	2.96
pH 5.0 + H_2_O_2_	0.92	0.37	0.92	0.06	0.98	3.32	0.94	5.57
pH 5.0 + GSH	0.92	0.42	0.91	0.06	0.99	3.83	0.93	7.05
pH 5.0 + GSH + H_2_O_2_	0.85	0.82	0.97	0.07	0.96	7.59	0.95	12.35
pH 5.0 + RF	0.87	0.37	0.97	0.06	0.97	3.40	0.96	4.85
pH 5.0 + H_2_O_2_ + RF	0.89	0.39	0.96	0.06	0.98	3.59	0.96	5.51
pH 5.0 + GSH + RF	0.91	0.50	0.97	0.05	0.99	4.55	0.98	6.38
pH 5.0 + GSH + H_2_O_2_ + RF	0.85	0.93	0.98	0.06	0.95	8.60	0.95	11.93

^a^Note: in the pH 7.4 + GSH group, the GSH concentration was 20 μM. In the pH 7.4 + H_2_O_2_ group, the H_2_O_2_ concentration was 100 μM. In the pH 7.4 + GSH + H_2_O_2_ group, the GSH concentration was 20 μM, and the H_2_O_2_ concentration was 100 μM. In the pH 5.0 + GSH/pH 5.0 + GSH + RF group, the GSH concentration was 10 mM. In the pH 5.0 + H_2_O_2_/pH 5.0 + H_2_O_2_ + RF group, the H_2_O_2_ concentration was 100 μM. In the pH 5.0 + GSH + H_2_O_2_/pH 5.0 + GSH + H_2_O_2_ + RF group, the GSH and the H_2_O_2_ concentration was 10 mM and 100 μM, respectively.

**Table 2:** Comparison of CUR-Fe NPs release models in vitro.^a^

Groups	*R*^2^ value for release							

	Zero-order model	*K*_0_	First-order model	*k*_1_	Higuchi model	*k*_H_	Ritger–Peppas model	*k*

pH 7.4	0.97	0.16	0.87	0.03	0.98	1.38	0.94	1.89
pH 7.4 + GSH	0.96	0.20	0.76	0.04	0.97	1.80	0.89	3.59
pH 5.0	0.99	0.28	0.59	0.05	0.97	2.39	0.75	5.71
pH 5.0 + GSH	0.94	0.75	0.88	0.06	0.99	6.79	0.94	13.02
pH 5.0 + RF	0.91	0.59	0.88	0.07	0.98	5.38	0.91	10.97
pH 5.0 + GSH + RF	0.95	0.95	0.88	0.05	0.99	8.51	0.93	15.18

^a^Note: in the pH 7.4 + GSH group, the GSH concentration was 20 μM. In the pH 5.0 + GSH/pH 5.0 + GSH + RF group, the GSH concentration was 10 mM.

### Cellular uptake study

**Figure 5:**
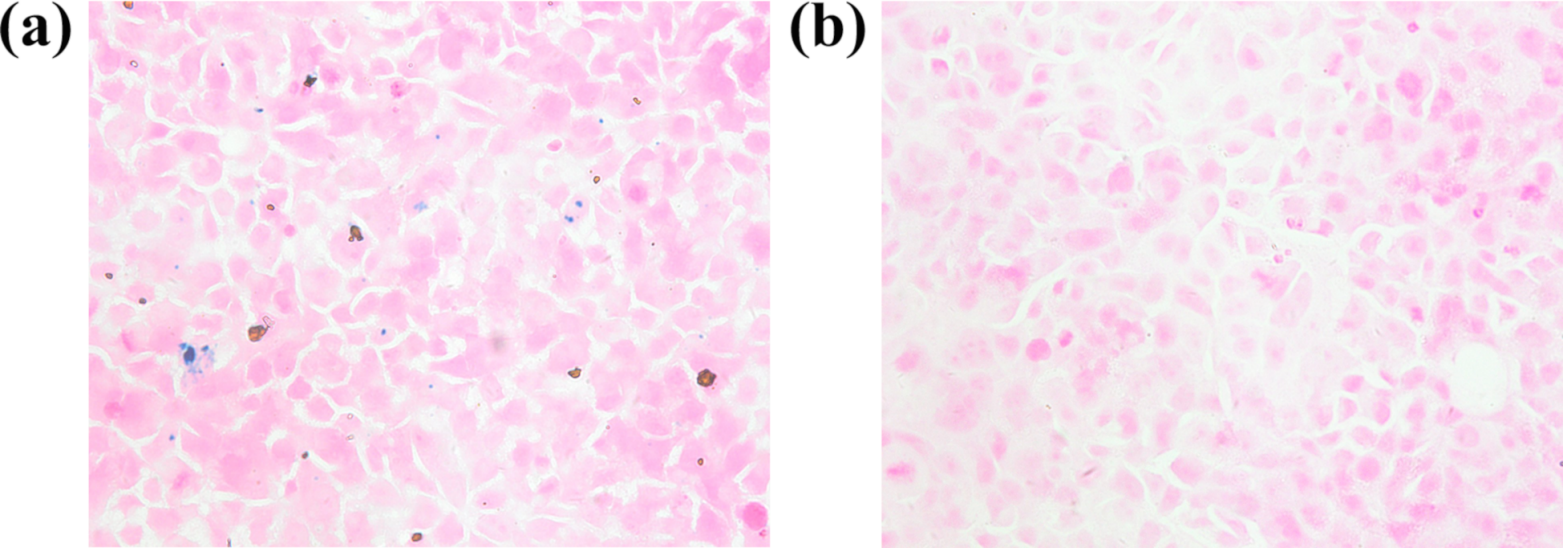
a) Prussian blue staining of Huh-7 cells in the NFs group. b) Prussian blue staining of Huh-7 cells in the control group.

Prussian blue staining was performed to detect the ability of Huh-7 cells to uptake NFs, as shown in Figure 5. Compared with those in the control group, blue particles were observed in the cytoplasm and extracellular space of Huh-7 cells after incubation with NFs for 24 h. These results indicated that cells can phagocytose NFs.

### Cytotoxicity of the NFs

**Figure 6:**
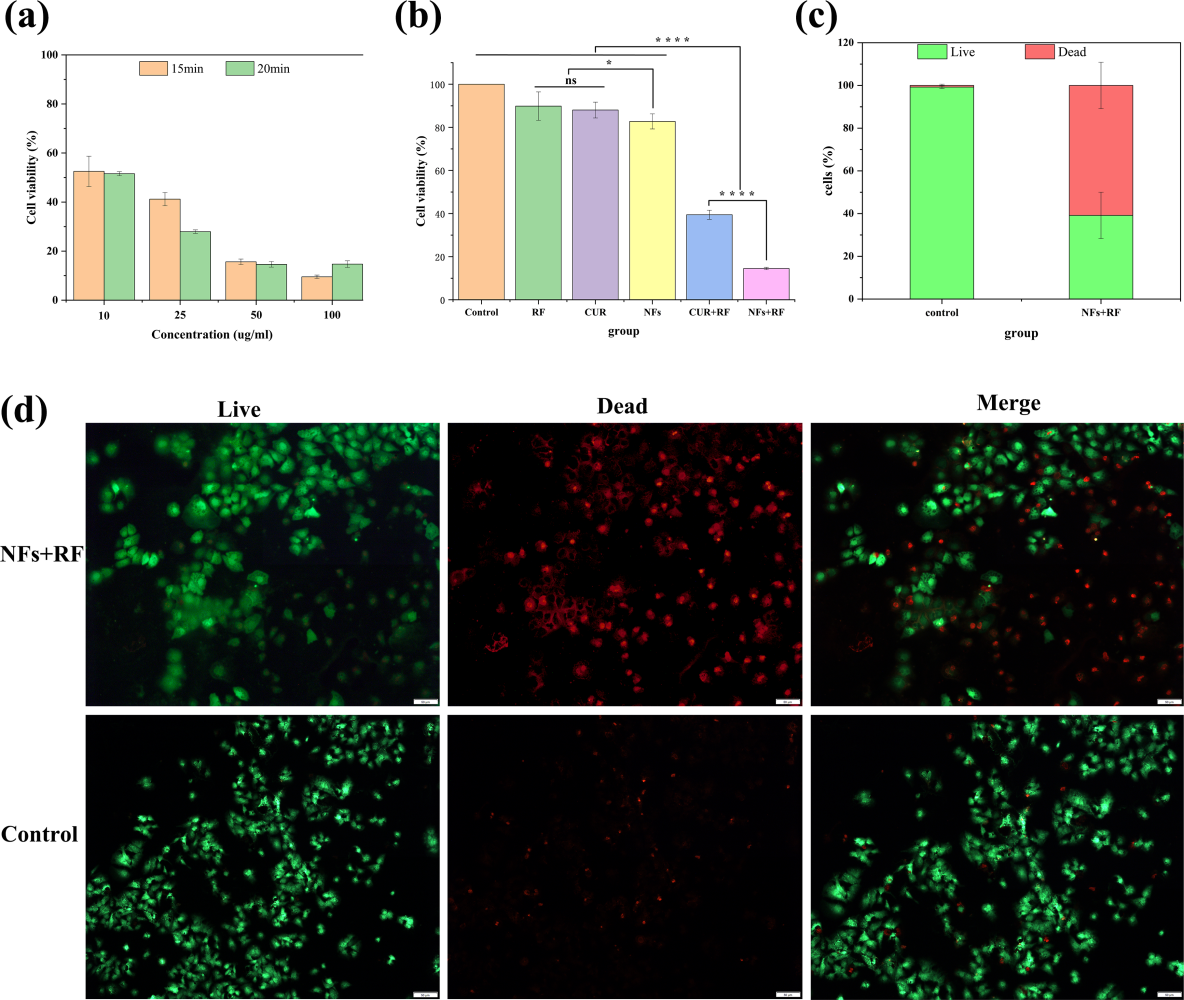
a) Toxicity of different concentrations of NFs to Huh-7 cells after heating for 15/20 min. b) Toxicity of different treatments (the concentration was 50 µg/mL for all samples) to Huh-7 cells after heating for 20 min (*p < 0.05, ****p < 0.0001, ns as not significant). c) Quantification of live/dead cells. d) Live/dead staining of NFs+RF and control groups after 4 h of incubation. (Scale bar: 50 μm).

Cell Counting Kit-8 (CCK-8) assays were performed to measure the viability of Huh-7 cells exposed to CUR-Fe@MnO_2_ NFs. The results showed that the optimal concentration of CUR-Fe@MnO_2_ NFs was 50 µg/ mL, and the optimal RF heating time to reach 41 ± 1 °C was 20 min (Figure 6a). The cytotoxicity of NFs was measured in normal liver cells (THLE-2 cells), and the cell viability rate was 105%, indicating that NFs had no significant toxic effects on normal liver cells. At the optimal concentration of NFs, the antitumor effects of the RF, CUR, and CUR-Fe@MnO_2_ NFs on Huh-7 cells were similar, which indicated that their toxicity to Huh-7 cells was limited. There was no significant difference in cell viability between RF, CUR, CUR-Fe@MnO_2_ NFs, and the control group (Figure 6b). Both CUR and CUR-Fe@MnO_2_ NFs exhibited high cytotoxicity after RF hyperthermia. Compared with that in the CUR-Fe@MnO_2_ NFs group, Huh-7 cell viability was 14.62% in the CUR-Fe@MnO_2_ NFs + RF group. These findings suggested that the antitumor effect of CUR-Fe@MnO_2_ NFs was significantly enhanced by RF hyperthermia.

To further test the cytotoxicity of the NFs group, we performed live and dead staining (Figure 6c,d). Dead cells were stained red and living cells were stained green. The results showed that the amount of red increased in the NFs+RF group, further indicating that RF hyperthermia could significantly enhance the antitumor effect of CUR-Fe@MnO_2_ NFs. However, there was no difference in cell morphology and viability in the control group. The reasons may be that (1) RF heating increased the degradation and release of CUR-Fe@MnO_2_ NFs and (2) RF heating increased the permeability of the cell membrane to improve the antitumor effects of CUR.

## Conclusion

In this study, responsive CUR-Fe@MnO_2_ NFs were successfully synthesized, and it was demonstrated that RF heating improved antitumor effect of NFs in vitro. The combination of RF heating responsive nanoflowers and dual-modal contrast agents for MRI (*T*_1_ and *T*_2_) may establish the foundation for HCC treatment.

## Experimental

### Materials

All chemical reagents were of analytical grade. Ferric chloride hexahydrate (FeCl_3_·6H_2_O), trisodium citrate (C_6_H_9_Na_3_O_9_), sodium acetate trihydrate (NaOAc·3H_2_O), potassium permanganate (KMnO_4_), acetonitrile, ethylene glycol (EG), triethylamine (TEA), curcumin (CUR), bis(4-hydroxyphenyl) disulfide (HPS), hexachlorocyclotriphosphazene (HCCP), polyethylene glycol (PEG) and 2-(*N*-morpholino) taurine (MES) were obtained from Shanghai Rhawn Chemical Technology Co., Ltd. (Shanghai, China). Ultrapure water was obtained from a Laibopate purification system (Changchun Lab Partner Technology Development Co., Ltd., Changchun, China) and used in all experiments. Human hepatocellular carcinoma cells (Huh-7 cells) and Dulbecco’s modified Eagle’s medium (DMEM, ZQ-101) were purchased from Shanghai Zhongqiao Xinzhou Biotechnology Co., Ltd. (Shanghai, China).

### Preparation of Fe_3_O_4_ NCs

Fe_3_O_4_ NCs were synthesized using a microwave hydrothermal synthesis method with a computer microwave ultrasonic synthesis/extraction instrument (XH-300A^+^, Beijing Xianghu Technology Development Co., Ltd., Beijing, China) [32–33]. First, trisodium citrate (0.40 g) was dissolved in pure EG (40 mL) and sonicated at room temperature until complete dissolution was achieved. Then, FeCl_3_·6H_2_O (1.35 g) was added with stirring. Next, NaOAc·3H_2_O (2.0 g) was added at room temperature with stirring for 30 min. The mixed solution was then put into a microwave reactor at 200 °C for 2 h. After the reactants cooled to room temperature, Fe_3_O_4_ NCs were obtained by magnetic separation followed by vacuum drying at 60 °C for 24 h.

### Synthesis of CUR-Fe NPs

Fe_3_O_4_ NCs (12 mg) were dispersed into acetonitrile (60 mL) with ultrasonication (XM-P06H, Xiaomei Ultrasonic Instrument Co., Ltd., Kunshan, China) for 30 min at room temperature. HCCP (15 mg), CUR (15.9 mg), and HPS (5.4 mg) were subsequently added with sonication for 30 min. TEA (2 mL) was dispersed into acetonitrile (10 mL), and this mixed solution was added dropwise to the above suspension for 3 min. The resulting mixture was subsequently sonicated for 4 h. Under the effect of TEA, the hydroxy groups on CUR and HPS became activated and replaced the chlorine atoms on HCCP, which led to the coassembly of the coating structure on the surface of Fe_3_O_4_ NCs. The generated CUR-Fe NPs were subsequently collected by magnetic separation and stored for the next step. In addition, the content of CUR in the supernatant was detected by a microplate reader (SPECTRAMAX190, Molecular Devices, USA) at 426 nm. DLE and DEE were calculated according to the following equations:


[1] $$\text{DLE}\left(\%\right)=\frac{m_{\text{added}}-m_{\text{supernatant}}}{m_{\text{NPs}}}\times 100\%$$

[2] $$\text{DEE}\left(\%\right)=\frac{m_{\text{added}}-m_{\text{supernatant}}}{m_{\text{added}}}\times 100\%,$$

where *m*_added_ is the mass of CUR added to prepare the NPs, *m*_supernatant_ is the mass of CUR in the supernatant, and *m*_NPs_ is the mass of the preparation after the completion of the NPs synthesis.

### Synthesis of CUR-Fe@MnO_2_ NFs

The prepared CUR-Fe NPs (9 mg) were suspended in 30 mL of deionized water and sonicated with KMnO_4_ powder (1.5 mg) for 10 min at room temperature to ensure that the NPs were well suspended. Then, MES (2.7 mg) and PEG (0.3 mg) were dispersed into 5 mL of water and added to the mixture dropwise for 5 min with ultrasonication (100 W, 40 kHz) at room temperature for 30 min [34]. The obtained CUR-Fe@MnO_2_ NFs were collected by magnetic separation and washed 3 times with deionized water.

### Characterization of the synthesized nanomaterials

The morphology and size of Fe_3_O_4_ NCs, CUR-Fe NPs, and CUR-Fe@MnO_2_ NFs were determined via transmission electron microscopy (Tecnai F20, FEI, USA). The TEM sample was added to ethanol and ultrasonically dispersed. Then the dispersed liquid was added dropwise to the copper net. After drying, the US FEI Tecnai F20 TEM was used at an accelerated voltage of 200 kV to capture the morphology in high resolution. Zeta potentials and hydrodynamic diameters were measured by a Malvern Zetasizer Nano ZS instrument (Nano ZS90, Malvern, UK). A 2 mg NF sample was added into 1 mL of deionized water and directly detected by ZS90. The detection angle was 90 degrees. The compositions were also analyzed using Fourier-transform infrared spectroscopy (Nicolet iS50, Thermo Scientific, USA) in the range of 400–4000 cm^−1^. The X-ray diffractometer patterns were characterized by the X-ray diffractometer (D8 ADVANCE, Bruker, Karlsruhe, Germany). The iron and manganese concentrations were determined using an inductively coupled plasma mass spectrometry (ICP‒MS) (7800, Agilent, Santa Clara, USA). The magnetic properties were evaluated on a Quantum Design PPMS-9T vibrating magnetometer at 300 K in a magnetic field from −3.0 T to 3.0 T (PPMS-9T, Quantum Design, San Diego, USA). Longitudinal (*T*_1_) and transversal (*T*_2_) relaxation times were measured on a 3.0 T MR scanner (MAGNETOM Skyra, Siemens Healthcare, Erlangen, Germany).

### Drug release

The release of CUR and Mn^2+^ from the CUR-Fe@MnO_2_ NFs was explored via dialysis. In brief, CUR-Fe@MnO_2_ NFs were released in PBS containing 0.7% (w/w) Tween 80 at different pH values (7.4 or 5.0) with or without GSH (20 μM/10 mM) and with or without H_2_O_2_ (100 μM), in which the pH 5.0 + GSH + H_2_O_2_ was used as the simulated TME. CUR-Fe@MnO_2_ NFs (2 mg/mL, 1 mL) were placed inside a dialysis membrane (MWCO of 3500 Da) and subsequently added to 100 mL of different PBS solutions at 37 °C in a thermostatic shaker at 100 rpm (Shanghai JINGQI Instrument Co., Ltd., China). Then, 2 mL of samples was collected from PBS at different time points (0, 4, 24, 48, and 72 h) and replenished with an equal volume of PBS. The absorbance of each of the samples was measured with a microplate reader at a wavelength of 426 nm to determine the concentration of CUR. The Mn^2+^ concentrations were determined using an ICP‒MS, and the changes in concentration were plotted. The process was repeated three times for each sample. The data were subsequently inserted into Origin 2021, and the plotted curves showed the in vitro release of CUR and Mn^2+^ from the CUR-Fe@MnO_2_ NFs.

To verify the Mn^2+^ and CUR release behavior from the CUR-Fe@MnO_2_ NFs after RF heating in vitro, CUR-Fe@MnO_2_ NFs in different environments were heated by RF for 20 min, after which dialysis was performed as described above.

Release profiles obtained for different pH buffer solutions were fitted to four different mathematical models used to determine the kinetics of drug release from delivery systems: zero order, first order, Higuchi, and Ritger–Peppas [35–36]. The model that exhibited the adjusted *R*-square closest to unity was selected as the best fit. The functions of the models considered are: zero-order model: ƒ_t_ = *K*_0_*t*, where ƒ_t_ is the fraction of CUR in NFs dissolved at time *t*, and *K*_0_ is the Zero order release constant. First-order model: *Q*_t_ = *Q*_0_exp(−*k*_1_*t*), where *Q*_t_ is the amount of CUR in NFs released at time *t*, *Q*_0_ is the initial amount of CUR in NFs, and *k*_1_ is First order rate constant. Higuchi Model: *Q*_t_ = *k*_H_*t*^1/2^, where *Q*_t_ is the amount of CUR in NFs released after time *t*, and *k*_H_ is the Higuchi dissolution constant. Ritger–Peppas Model: *M*_t_/*M*_∞_ = *kt*^n^, where *M*_t_/*M*_∞_ is the fraction of CUR in NFs released at time *t*, *k* is the rate constant, and n is the diffusional release exponent.

### Cellular uptake of CUR-Fe@MnO_2_ NFs

The CUR-Fe@MnO_2_ NFs uptake by Huh-7 cells was observed under a fluorescence microscope. Huh-7 cells were incubated with 50 µg/mL NFs for 24 h. Then, 4% paraformaldehyde was used to fix the Huh-7 cells for 15 min, followed by washing with PBS. Fixed cells were stained by the Prussian Blue kit (Solarbio Life Sciences, Beijing, China) at 37 °C for 30 min.

### Cytotoxicity assays

The Huh-7 cells were seeded onto 4-chamber cell culture slides (Nalge Nunc International, Rochester, NY, USA) at a density of 1.0 × 10^6^ cells/chamber. The cells were incubated at 37 °C in 5% CO_2_ for 24 h. The DMEM medium was replaced with fresh medium containing various concentrations of CUR-Fe@MnO_2_ NFs (10, 25, 50, and 100 µg/mL), and the 4-chamber cell culture slides were placed in a 37 °C water bath. Then, a 0.035 inch heating guidewire (HG) was attached to the bottom of the first of the 4-chamber cell culture slides and connected to a 180 MHz custom-made radiofrequency generator [37]. When the RF generator was operated through the HG at 2~3 W, the temperature in the first chamber increased to 41 ± 1 °C for 15 and 20 min, respectively. After another 4 h of incubation, cell viability was measured by a CCK-8 assay. The temperature of each chamber was recorded by a 0.9 mm optical fiber temperature probe (FL-2000, Anritsu Meter Co., Ltd., Tokyo, Japan).

The 2.0 × 10^5^ and 1.0 × 10^6^ Huh-7 cells were seeded onto 96-well plates or 4-chamber cell culture slides, and incubated at 37 °C in 5% CO_2_ for 24 h. According to the above results, the optimal concentration of NFs was determined. The cells were divided into different groups (control group, RF, CUR, CUR-Fe@MnO_2_ NFs, CUR + RF, and CUR-Fe@MnO_2_ NFs + RF), and after 4 h of incubation, cell viability was determined by a CCK-8 assay.

The cell viability was calculated according to the following equation:


[3] $$\text{Cell viability}\left(\%\right)=\frac{\text{treatment group}}{\text{control group}}\times 100\%.$$

The THLE-2 cells (1.5 × 10^4^) were seeded onto 96-well plates and incubated at 37 °C in 5% CO_2_ for 24 h. The cell medium was replaced either with fresh medium or medium supplemented with NFs, and the cells were cultured for 4 h. The cell viability was determined by a CCK-8 assay. Three independent repetitions were performed in each group.

The cytotoxicity of the NFs+RF group was also observed by live/dead staining. Huh-7 cells (1.0 × 10^6^) were seeded onto 4-chamber cell culture slides, and incubated at 37 °C in 5% CO_2_ for 24 h. The medium was replaced either with fresh medium or medium supplemented with NFs (the group with NFs was heated for 20 min) and the cells were cultured for 4 h. The staining was performed with calcein-AM and ethidium homodimer 1 (EthD-1). Images of the stained cells were collected using a fluorescence microscope (OLYMPUS microscope, Tokyo, Japan) after live/dead staining.

### In vitro magnetic resonance imaging

CUR-Fe@MnO_2_ NFs can release Mn^2+^ and Fe_3_O_4_ NCs. Mn^2+^ can shorten the *T*_1_ effect, and Fe_3_O_4_ NCs can shorten the *T*_2_ effect. In this study, CUR-Fe@MnO_2_ NFs at pH 5.0 + GSH + H_2_O_2_ were mixed with a 1% agarose solution to create solutions of different concentrations of NFs (0, 2, 4, 6, and 8 mM), which were subsequently placed into 1.5 mL Eppendorf tubes. The samples were then placed in a 3.0 T MRI scanner to determine the longitudinal relaxation time (*T*_1_) and the transversal relaxation time (*T*_2_). The images were acquired by *T*_1_/*T*_2_ mapping sequences using the head coil. The parameters of the *T*_1_ mapping sequences were as follows: repetition time (*T*_R_), 7.02 ms; echo time (*T*_E_), 1.87, 2.1, 2.45, 2.75, and 3.69 ms; field of view (FOV), 234 × 300 mm; matrix, 280 × 512; slice thickness, 1.5 mm; and bandwidth, 260 Hz/Px. The parameters of the *T*_2_ mapping sequences were as follows: *T*_R_ 1220 ms; *T*_E_, 13.8, 27.6, 41.4, 55.2, and 69 ms; FOV, 159 × 159 mm; matrix, 384 × 384; slice thickness, 3 mm; and bandwidth, 228 Hz/Px. Finally, *T*_1_/*T*_2_ was calculated and concentration/relaxation rate curves were constructed.

### Statistical analysis

Statistical Package for the Social Sciences (SPSS 26.0, IBM, USA) was used for statistical analysis. All data are displayed as the mean ± standard deviation (SD). Data analysis was performed using one-way analysis of variance (ANOVA). For all comparisons, if *p* < 0.05, the difference was considered statistically significant and was denoted as, *p < 0.05, ****p < 0.0001, ns (not significant).

## Data Availability

The data that supports the findings of this study is available from the corresponding author upon reasonable request.
